# Complete genome of a *Serratia* species isolated from PFAS-impacted soil

**DOI:** 10.1128/MRA.00640-23

**Published:** 2023-11-17

**Authors:** Isabel R. Baker, Sophie M. Colston, William J. Hervey, Lina J. Bird

**Affiliations:** 1 Center for Bio/Molecular Science and Engineering, US Naval Research Laboratory, Washington, DC, USA; University of Delaware College of Engineering, Newark, Delaware, USA

**Keywords:** pollution, soil microbiology, PFAS, *Serratia*

## Abstract

*Serratia* sp. B1 is a bacterial species isolated from soil highly impacted by perfluoroalkyl and polyfluoroalkyl substances, a family of biopersistent contaminants colloquially known as “forever chemicals.” Here, we report the genome of *Serratia* sp. B1, sequenced with Oxford Nanopore Technology. The genome consists of one 5.14 Mbp chromosome and one 92 kb plasmid.

## ANNOUNCEMENT

Perfluoroalkyl and polyfluoroalkyl substances (PFAS) are fluorinated chemicals that have been manufactured for various commercial applications since the 1950s (1). Due to their stable carbon-fluorine bonds, these compounds are now pervasive in soil and water and persist in the biota that interface with these resources (1). Here, we report the genome of a bacterium, *Serratia* sp. B1, which was isolated from soil highly impacted by PFAS and whose growth is unaffected by perfluooctanoic acid (PFOA) at concentrations 2 million times higher than the proposed Environmental Protection Agency (EPA) maximum contaminant level (2).

We isolated *Serratia* sp. B1 from PFAS-impacted, fine sand soil (10–15 feet below ground) in the mid-Atlantic region of the United States in September 2022; soil and groundwater were collected with direct-push technology drilling. A total of 500 µL of a 0.1 g/ml soil/groundwater slurry was transferred to 5 mL of filter-sterilized groundwater spiked with 1 ppm PFOA (Aldrich) and 1 g/L yeast extract. After shaking at 18°C for 1 week, cultures were passaged into Reasoner’s 2A liquid broth (R2A, Teknova) spiked with 10 ppm PFOA. Following 1 week of shaking at 18°C, an aliquot of the culture was swabbed onto a 10 ppm PFOA and R2A agar plate and incubated at 30°C. After 24 h, the plate was speckled with opaque colonies that were pink or cream in color. A cream-colored colony was selected and swabbed onto a fresh plate; this process was repeated until streaking produced uniform, cream-colored colonies. One colony was selected to inoculate fresh R2A, and the resulting culture was then aliquoted into 25% glycerol stocks frozen at −80°C.

For sequencing, the isolate was cultured in R2A for 16 h with shaking at 30°C. DNA was extracted using the MasterPure Complete DNA and RNA Purification Kit (Lucigen). Libraries were prepared with the Oxford Nanopore Technologies Rapid Barcoding Kit (SQK-RBK004). Long-read sequencing was performed using the MinION Mk-1C with a FLO-MIN106D R9 flow cell (Oxford Nanopore Technologies). A total of 125,849 raw reads were basecalled and demultiplexed using Guppy (v.6.4.6), with an average length of 14,835 bp. Genome assembly with Flye version 2.9.2 (3) revealed two circular contigs—a 5,139,913 bp chromosome with 59.54% G+C content and a 92,029 bp plasmid with 52.78% G+C content. Final genome coverage was 625× and 3,292× for the chromosome and plasmid, respectively. CheckM analysis (4) indicated an estimated genome completeness of 98.767% with 0% contamination. All software was run with default parameters.

Taxonomic identity was assessed as part of the National Center for Biotechnology Information (NCBI) bacterial genome submission process, which indicated that *Serratia* sp. B1 is likely a strain of *Serratia ureilytica* (average nucleotide identity of 98.66% with *S. ureilytica* CCUG:50595). The genome was annotated with the NCBI Prokaryotic Genome Annotation Pipeline version 6.5. The chromosome contains 4,932 coding sequences, 22 rRNAs, and 89 tRNAs. The plasmid contains 98 coding sequences. The proportion of frameshifted pseudogenes (737/793 pseudogenes) in this genome is a characteristic of non-hybrid assemblies generated exclusively with Nanopore reads, which often indicates a higher error rate. Nevertheless, this genome is a valuable resource for understanding the broader impacts of PFAS on our ecosystem and could inform future remediation efforts.

## Data Availability

The chromosome and plasmid sequences of *Serratia* sp. B1 have been deposited in GenBank under accession numbers CP123616 and CP123617, respectively, under NCBI BioProject PRJNA956891. MinION raw data are available in the NCBI Sequence Read Archive under accession numbers SRR24895934.
